# Molecular Determinants Elucidate the Selectivity in Abscisic Acid Receptor and HAB1 Protein Interactions

**DOI:** 10.3389/fchem.2020.00425

**Published:** 2020-06-04

**Authors:** Jing-Fang Yang, Chun-Yan Yin, Di Wang, Chen-Yang Jia, Ge-Fei Hao, Guang-Fu Yang

**Affiliations:** ^1^State Key Laboratory Breeding Base of Green Pesticide and Agricultural Bioengineering, Key Laboratory of Green Pesticide and Agricultural Bioengineering, Ministry of Education, Research and Development Center for Fine Chemicals, Guizhou University, Guiyang, China; ^2^School of Life Science, Wuchang University of Technology, Wuhan, China; ^3^Key Laboratory of Pesticide & Chemical Biology, Ministry of Education, College of Chemistry, Central China Normal University, Wuhan, China; ^4^International Joint Research Center for Intelligent Biosensor Technology and Health, Central China Normal University, Wuhan, China; ^5^Collaborative Innovation Center of Chemical Science and Engineering, Tianjin, China

**Keywords:** abscisic acid, PYR/PYL/RCARs, pyrabactin, selectivity, molecular dynamics

## Abstract

The abscisic acid (ABA), as a pivotal plant hormone, plays a key role in controlling the life cycle and adapting to the environmental stresses. The receptors of ABA are the Pyrabactin resistance/Pyrabactin resistance-like/regulatory component of ABA receptors (PYR/PYL/RCAR, PYLs for simplicity), which regulate the protein phosphatase 2Cs (PP2Cs) in the signal pathway. As an important ABA-mimicking ligand, Pyrabactin shows the activation function to parts of members of PYLs, such as PYR1 and PYL1. Due to the antagonism of Pyrabactin to PYL2, it was used as a probe to discover a part of ABA receptors. Since then, many researchers have been trying to find out the determinants of the selective regulation of PYLs and PP2Cs interaction. However, the roles of residues on the selective regulation of PYR1/PYL2 and PP2Cs interaction induced by Pyrabactin are still ambiguous. This research investigated the selective activation mechanism of Pyrabactin through the sequence alignment, molecular docking, molecular dynamics simulation, and binding free energy calculation. Furthermore, the electrostatic and hydrophobic interaction differences induced by Pyrabactin and agonists were compared. The results indicate that Leu137/Val114, Ser85/Ser89, and Gly86/Gly90 from the pocket and gate of PYR1/PYL2 are the vital residues for the selective activation of Pyrabactin. Meanwhile, the electrostatic interaction between PP2Cs and PYLs complexed with agonists was improved. This mechanism provides strong support for the design of selective agonists and antagonists.

## Introduction

Drought stress is a severe abiotic stress, which may lower the crop yield of the world (Vishwakarma et al., 2017; Zhang et al., 2018). The phytohormone abscisic acid (ABA), as an antistress regulator, has aroused widespread concern (Park et al., 2009). Indeed, it not only regulates many aspects of plant growth and development but also responds to environmental stresses (e.g., drought, salinity, cold, and UV radiation) (Verma et al., 2016). Generally speaking, ABA-deficient plants show defects in stomatal regulation, seed dormancy, and germination (Finkelstein, 2013). Due to the essentiality of ABA in the plants, the discoveries of ABA receptor protein and the core signaling complexes may transmit cues for the understanding of subsequent molecular events and plant antistress phenotype (Sah et al., 2016).

The bona fide ABA receptor and the regulation mechanism were discovered and understood in 2009 (Ma et al., 2009; Park et al., 2009; Pennisi, 2009). The receptors of ABA are Pyrabactin resistance/Pyrabactin resistance-like/regulatory component of ABA receptors (PYR/PYL/RCARs, simplified as PYLs). There are 14 members in *Arabidopsis thaliana*, consisting of PYR1, PYL1–PYL13 (Yang et al., 2019). Besides, PYLs contain a binding pocket with a loop as gate, and it is closed in response to ABA (Melcher et al., 2009), which will create a binding surface outside the gate loop for protein phosphatase 2Cs (PP2Cs), including ABI1, ABI2, HAB1, HAB2, and PP2CA. Then, SNF1-related protein kinase 2s (SnRK2s) are activated to phosphorylate downstream effectors (Yamaguchi-Shinozaki and Shinozaki, 2006; Melcher et al., 2009). As a gate-lock mechanism, it offers a key clue for the discovery of ABA mimic molecule and plays a pivotal role in revealing some specific regulation pathways.

ABA mimic molecules, which function as agonists or antagonists of PYLs and have the potential application in agriculture, may deepen the knowledge of the signaling and promote the study of the ABA receptor. Hence, there is a wide interest in the discovery of ABA mimic molecules. Pyrabactin, an early synthetic ABA mimic, functions as an agonist of PYR1 and PYL1 but as an antagonist of PYL2 (Park et al., 2009; Yuan et al., 2010). It is sufficient to activate ABA responses in seeds but yields a minimal response in vegetative tissues and cannot make plants survive from drought tolerance (Okamoto et al., 2013). Another selective agonist toward a part of PYLs (PYR1, PYL1-PYL3, and PYL5), Quinabactin (also known as AM1), is a promising agrochemical to elicit stomatal closure and enhance crop drought resistance (Cao et al., 2013; Okamoto et al., 2013). After structure optimization, the fluoro-substitution compound (AMF4) was synthesized. AMF4 has a long-lasting effect to promote the stomatal closure, induce the expression of stress-responsive genes, and activate the same PYLs as AM1 (Cao et al., 2017). Overall, Pyrabactin is the only selective agonist of PYR1 and PYL2, and it is rotated for 90° in PYL2 relative to that in PYR1, which is not found for AM1 and AMF4. Because of the significance of Pyrabactin for the selective activation of PYLs, a lot of research have focused on its distinct selectivity to PYLs. It is believed that some important amino acids, such as Val67 and Val114 of PYL2, are important for the selectivity (Peterson et al., 2010; Yuan et al., 2010). However, the dynamics roles and the energy determinants of the residues on the PYLs–PP2Cs interface remain unclear, which are crucial for understanding the selectivity and performing precise ABA mimic molecule design.

Therefore, in this study, the complex structures of HAB1 and PYR1/PYL2 with ABA, AM1, AMF4, and Pyrabactin binding were used to perform molecular simulation and reveal the selective activation determinants of PYLs. Additionally, the sequence alignment, structures comparison, molecular dynamics (MD) simulation, and binding free energy and decomposition were performed based on public tools and our protocols (Hao et al., 2016; Yang et al., 2020). Beyond that, the interactions between PYR1/PYL2 and HAB1 were analyzed and decomposed. Finally, it is found that the electrostatic (ELE) and hydrophobic interactions between PYR1/PYL2 and HAB1 induced by agonists are conserved. At the same time, pocket residues Leu137/Val114 and gate residues Ser85/89 and Gly86/90 of PYR1/PYL2 are the key residues for selectivity of Pyrabactin. More importantly, this finding provides guidance for the design of agonists and antagonists of PYLs.

## Method

### Sequences Alignment

There are already a large number of tools available to analyze sequences and structures, such as ClustalW (Thompson et al., 1994), Muscle (Edgar, 2004), T-coffee (Di Tommaso et al., 2011), and Dialign (Al Ait et al., 2013). Discovery Studio is a popular commercial software to perform combined analyses. Therefore, 14 *Arabidopsis thaliana* PYLs sequences downloaded from the National Center for Biotechnology information (NCBI) were aligned using an automatic tool in Discovery Studio 2.5 (BIOVIA Discovery Studio, 2009).

### Molecular Docking and Structures Preparation

The crystal structures of PYR1 (ABA)-HAB1 (PDB ID: 3QN1) (Dupeux et al., 2011), PYL1 (Pyrabactin)-ABI1 (PDB ID: 3NMN) (Melcher et al., 2010), PYL2 (ABA)-HAB1 (PDB ID: 3KB3) (Melcher et al., 2009), PYL2 (AM1)-HAB1 (PDB ID: 4LA7) (Okamoto et al., 2013), and PYL2 (AMF4)-HAB1 (PDB ID: 5VSR) (Cao et al., 2017) downloaded from the Protein Data Bank (PDB) database were superimposed and analyzed by Pymol software (Berman et al., 2000; DeLano, 2002), some of which were used for molecular docking and MD.

AutoDock 4.2 was applied to molecular docking (Morris et al., 2009). In this study, AM1, AMF4, and Pyrabactin were docked into the crystal structure of PYR1-HAB1 (PDB ID: 3QN1) (Yuan et al., 2010) to generate initial structures for MD. The Lamarckian genetic algorithm (LGA) was applied for the conformational search of the ligand (Morris et al., 1998). The grid size was set as 40 ×40 ×40, and the grid space was set to the default value of 0.375 Å. A total of 256 runs were launched for each compound. Most of the parameters for the docking calculation were set to the default values recommended by the software.

To reveal the conformational selective mechanism of Pyrabactin to PYR1 and PYL2, the structures of PYR1 (Pyrabactin)-HAB1 from docking and crystal structure PYL2 (Pyrabactin) (PDB ID: 3NS2) were superimposed. Then, the Pyrabactin was extracted from two complexes and saved with the other ABA receptor to get the complexes PYR1 (Pyrabactin) and PYL2 (Pyrabactin) with the initial Pyrabactin structure from PYL2 (Pyrabactin) and PYR1 (Pyrabactin)-HAB1, respectively. Meanwhile, HAB1 was saved with the PYL2 (Pyrabactin) to build up the initial structure of PYL2 (Pyrabactin)-HAB1 for later MD.

### MD Simulation and Trajectory Analysis

MD simulations were performed using the AMBER 16 software package with the ff14SB force field (Case et al., 2016). The ligand electrostatic potentials were computed at the HF/6-31G^*^ level in the Gaussian 03 program (Frisch et al., 2004). The RESP fitting technique in AMBER was used to determine the partial charges (Wang et al., 2000). The force-field parameters for the ligands were generated with the general AMBER force field (gaff) by the Antechamber program (Case et al., 2005). Each complex was immersed in a cubic box of the TIP3P water model with an 8.0-Å minimum solute-wall distance. Na^+^ or Cl^−^ ions were added to neutralize each complex system.

The complex systems were optimized before the simulation as follows. First, the movement was allowed only for hydrogen atoms. Next, the side chains were relaxed. Finally, all atoms were permitted to move freely. In each stage, energy minimization was executed by the steepest descent method for the first 1,000 steps and the conjugate gradient method for the subsequent 2,000 steps. After that, the systems were set up to obtain stable MD trajectories. Complex systems were gradually heated from 10 to 300 K in 200 ps, and more than 500 ps equilibrating calculation was executed at 1 atm and 300 K with applying periodic boundary conditions in the NPT ensemble to avoid edge effects. The 8 ns MD simulation of each system was performed. The snapshots extracted at every picosecond of the stable interval from the last 6 ns production MD trajectory using the CPPTRAJ module of AMBER were used for structural and energetic analysis. Meanwhile, the snapshots from the last 6 ns MD simulation processed were used to detect hydrogen bonds. The systems and timescales for all molecular dynamics simulations are listed in the Table S1.

### Binding Energy and Decomposition Calculation

For each snapshot, the binding energy (Δ*H*) of the protein (ligand)–protein complex was calculated by the molecular mechanics/generalized born surface area (MM/GBSA) method as in the following equation (Genheden and Ryde, 2015).

(1) $$\begin{array}{l} \Delta G_{\text{bind}}=\Delta E_{\text{MM}}+\Delta G_{\text{solv}}-T\Delta S \end{array}$$

(2) $$\begin{array}{l} \Delta E_{\text{MM}}=\Delta E_{\text{ele}}+\Delta E_{\text{vdw}}+\Delta E_{\text{int}} \end{array}$$

(3) $$\begin{array}{l} \Delta G_{\text{solv}}=\Delta G_{\text{GB}}+\Delta G_{\text{np}} \end{array}$$

The binding free energy (Δ*G*_bind_) equals the changes in the molecular mechanics component in gas phase (Δ*E*_MM_), solvation free energy (Δ*G*_solv_), and entropic contribution (–*T*Δ*S*). The molecular mechanics free energy (Δ*E*_MM_) is further split into electrostatic (Δ*E*_ele_), van der Waals (Δ*E*_vdw_), and bond, angle, and dihedral (Δ*E*_int_) energies. The solvation free energy (Δ*G*_solv_) can be divided into electrostatic solvation free energy (Δ*G*_GB_) and a nonpolar solvation free energy (Δ*G*_np_). The Δ*G*_GB_ to the solvation energy is computed with a GB module of the AMBER suite.

The decomposition analysis was also performed by mm_pbsa module of AMBER. The detailed procedure was described by Gohlke et al. (2003).

## Results and Discussion

### Sequence and Structure Comparison of PP2Cs Binding Domain of PYLs

**Figure 1:**
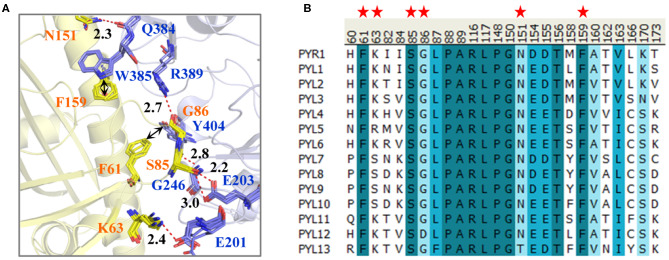
**(A)** The interactions on the binding surface of PYLs and HAB1 and **(B)** the multiple sequence alignment of 14 *Arabidopsis thaliana* PYLs. **(A)** The crystal structures of PYR1 (ABA)-HAB1 (PDB ID: 3QN1), PYL1 (Pyrabactin)-ABI1 (PDB ID: 3NMN), PYL2 (ABA)-HAB1 (PDB ID: 3KB3), PYL2 (AM1)-HAB1 (PDB ID: 4LA7), and PYL2 (AMF4)-HAB1 (PDB ID: 5VSR) were superimposed. The PYLs and PP2Cs are colored in yellow and blue, respectively. The residue numbers of PYLs and PP2Cs are labeled according to them in PYR1 and HAB1. **(B)** The important residues of PYLs on the binding surface are labeled with red stars.

To compare the binding surfaces of different PYLs and PP2Cs, we collected the PYLs–PP2Cs complex structures, including PYR1 (ABA)-HAB1 (PDB ID: 3QN1) (Dupeux et al., 2011), PYL1 (Pyrabactin)-ABI1 (PDB ID: 3NMN) (Melcher et al., 2010), PYL2 (ABA)-HAB1 (PDB ID: 3KB3) (Melcher et al., 2009), PYL2 (AM1)-HAB1 (PDB ID: 4LA7) (Okamoto et al., 2013), and PYL2 (AMF4)-HAB1 (PDB ID: 5VSR) (Cao et al., 2017). Subsequently, the collected structures were superimposed and compared (Figure 1A). Take PYR1-HAB1 as an example; Ser85 of PYR1 forms hydrogen bonds with Gly246 and Glu203 of HAB1. Meanwhile, Lys63, Gly86, and Asn151 of PYR1 form ELE interaction with Glu201, Arg389, and Gln384 of HAB1, respectively. In addition to hydrogen bonds, there is a *T*–π interaction between Phe61 of PYR1 and Tyr404 of HAB1 as well as a π–π interaction between Phe159 of PYR1 and Trp385 of HAB1. These interactions almost exist in all the complexes, except for the hydrogen bond between Lys90 of PYL1 (corresponding to Lys63 of PYR1) and Glu140 of ABI1 (Glu201 of HAB1). However, the long-range electrostatic interaction between them still exists. In a word, the interactions on the binding surface are very conservative.

For further exploring the conservativeness of *A. thaliana* PYLs and PP2Cs, the multiple sequence alignment of 14 *A. thaliana* PYL sequences was performed (Figure 1B). It could be found that the residues on the position of Phe61, Ser85, and Phe159 of PYR1 are highly conservative. Gly86 and Asn151 of PYR1 are replaced by Asp and Thr in PYL12 and PYL13, respectively. Nonetheless, the side chain of Gly86 does not influence the interaction between PYLs and PP2Cs shown by the binding mode. The least conservative site is the position of Lys63 of PYR1, which is Lys in PYR1, PYL1–PYL4, PYL6, and PYL11–PYL13, but Ser in PYL7–PYL10. Through comparison, we found that the binding surfaces of *A. thaliana* PYLs are in high conservation, especially for PYR1 and PYL1–PYL3.

### Comparison of the Binding Models of Pyrabactin in PYR1 and PYL2

It has been found that the interactions on the PYLR1/PYL2-PP2Cs binding surface are conservative. How does the Pyrabactin induce the selectivity? The X-ray crystal structures show that there were two absolutely different conformations for Pyrabactin in PYR1/PYL1 and PYL2 (Yuan et al., 2010). Apparently, PYL2 binding with Pyrabactin is insensitive to PP2Cs. However, PP2C is inhibited by PYR1 (binding with Pyrabactin) effectively. In other words, the influence of the conformation of Pyrabactin is significant. Therefore, four complexes, constructed based on the crystal structures PYR1 (PDB ID: 3QN1) and PYL2 (PDB ID: 3NS2) as well as the initial Pyrabactin structure from PYR1 (Conf1) and PYL2 (PDB ID: 3NS2, Conf2), were used to perform MD simulations in order to compare the binding models of Pyrabactin in atomic level (Table S1).

To verify the equilibration of the systems, the atomic root mean square deviations (RMSD) were calculated, and the convergences of energies were analyzed (Knapp et al., 2011; Dawson and Gygi, 2018). As displayed in the RMSD plots, the RMSDs of all the systems reach a certain value (Figure S1). It seems that there was no big conformational change of PYL2 complexed with Pyrabactin in two starting conformations (Figure S1). However, the RMSD values of the backbone of PYR1 and the heavy atoms of Pyrabactin with the initial structure in the PYL2 were much higher than those of the other three systems, indicating that the conformations of PYR1 and Pyrabactin were changed in this system (Figure S1B). In terms of the energy, the standard deviations (STDs) of binding free energy of these systems were low ranging from 1.01 to 1.61 kcal mol^−1^ (Table S2). All these results suggest that the systems had already reached equilibrations, and these trajectories may be used for further analysis.

**Figure 2:**
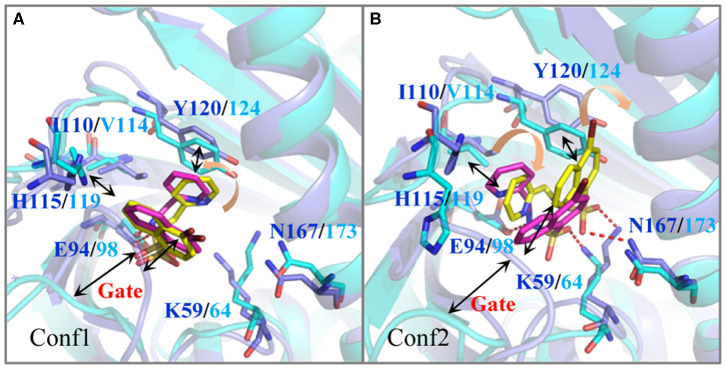
The comparison of the binding modes of Pyrabactin in PYR1 and PYL2 after MD. The starting structures of Pyrabactin in the PYR1 (Conf1, **A**) and PYL2 (Conf2, **B**) complexes were used. The PYR1 and PYL2 are colored in blue and cyan, and the corresponding Pyrabactin are colored in yellow and magentas.

For further revealing the impact of the initial structure of Pyrabactin on PYR1 and PYL2, the binding free energies and binding modes of these systems were compared. As we all know, MM/GBSA is a powerful tool in drug design to rank the binding affinities for systems without metals (Wang et al., 2019). Therefore, it was applied in this study. To be specific, the binding free energy of Pyrabactin in Conf1 (−20.08 kcal mol^−1^) was lower than that of Pyrabactin in Conf2 (−18.12 kcal mol^−1^) with PYR1. Meanwhile, the binding affinity of Pyrabactin in Conf2 (−13.33 kcal mol^−1^) is higher than the other one (−6.82 kcal mol^−1^, Table 1) with PYL2. Therefore, the Conf1 and Conf2 were favored conformations in the pockets of PYR1 and PYL2, respectively. Beyond that, the binding free energies of Pyrabactin and PYR1 were lower than those of Pyrabactin and PYL2 (Figure 2). Besides, the closed gate improved the Δ*E*_vdw_ and reduced the influence of Δ*G*_solv_. For Conf1, it did not undergo a big conformation change in PYL2 after the MD. There was a direct hydrogen bond between the sulfonamide of Pyrabactin and Glu94/98 of PYR1/PYL2. Furthermore, the naphthalene and pyridine of Pyrabactin formed the *T*–π and π–π interaction with His115/119 and Tyr120/124, respectively (Figure 2A). The only difference was that the pyridine of Pyrabactin moved near to the Val114 of PYL2 because of the short chain of Val, which induced the naphthalene to move always from the gate. As indicated by the result, it was hard for Pyrabactin in Conf1 to induce the closure of the gate of PYL2. For the Pyrabactin in Conf2, there was a deflection in PYR1. The longer side chain of Ile110 of PYR1 conflicted with the pyridine of Pyrabactin, which was pushed near to the Asn167 and Tyr120 (Figure 2B). This deflection made the protein outward and improved the penalty of Δ*G*_solv_, inducing the low binding affinity of Pyrabactin in Conf2 with PYR1. The hydrogen bond with Lys59/64 of PYR1/PYL2 remained, while the hydrogen bond with Glu98 of PYL2 was replaced by Asn167 of PYR1. These hydrogen bonds improved the Δ*E*_ele_ of this conformation both in PYR1 and PYL2. Meanwhile, the van der Waals (VDW) interaction between the naphthalene and pyridine of Pyrabactin and His115/119 and Tyr120/124 of PYR1/PYL2 stabilized the binding modes (Figure 1B). Apparently, the Pyrabactin in Conf2 disordered the structure of PYR1 and broke the active conformation of PYR1. At the same time, Pyrabactin was far from the closed gate in this binding mode, indicating that it is hard to induce the active conformation of PYL2.

**Table 1:** The binding free energy (kcal/mol) of Pyrabactin in Conf1 and Conf2 and PYLs (PYR1 and PYL2).

	**Δ*E*_**ele**_**	**Δ*E*_**vdw**_**	**Δ*E*_**MM**_**	**Δ*G*_**solv**_**	**Δ*G*_**cal**_**
PYR1-Pyrabactin (Conf1)	−16.18	−39.75	−55.93	35.86	−20.08
PYR1-Pyrabactin (Conf2)	−20.12	−39.51	−59.63	41.51	−18.12
PYL2-Pyrabactin (Conf1)	−15.00	−36.12	−51.12	44.30	−6.82
PYL2-Pyrabactin (Conf2)	−23.57	−37.09	−60.66	47.34	−13.33

Therefore, the different residue Ile110/Val114 is the determinant for the conformational selectivity of Pyrabactin in PYR1 and PYL2, which is consistent with the previous experimental data. The V114I mutant of PYL2 is able to inhibit the phosphatase activity of ABI1 in the presence of Pyrabactin just like PYR1 (Peterson et al., 2010; Yuan et al., 2010). This single residue alteration influences the Pyrabactin conformation in the pockets of PYLs, directly determining the state of the gate and the function of PYLs.

### Selective Activation Mechanism of PYR1 and PYL2

**Figure 3:**
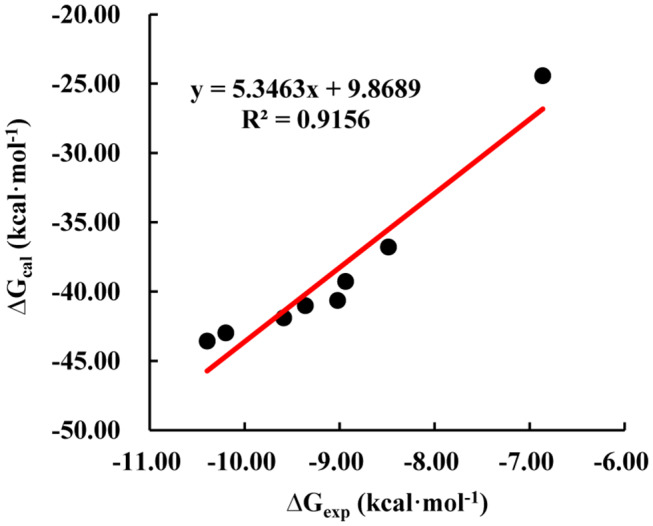
The correlation of calculated (Δ*G*_cal_) and experimental (Δ*G*_exp_) binding free energy.

In order to obtain dynamics conformation, we applied MD simulation on eight systems, including HAB1 complexed with PYR1 and PYL2 binding with ABA, AM1, AMF4, and Pyrabactin, respectively. Moreover, RMSD value per picosecond and binding energy per nanosecond in the last 6 ns were calculated to explore the dynamic stability of eight systems. In this process, the RMSD values of the backbone of PYR1/PYL2-HAB1 and the heavy atoms of ligands were lower than 2.5 and 0.5 Å (Figure S2). With regard to the binding energy, all the STDs were lower than 2.21 kcal mol^−1^ (Table S3). These results revealed that all the systems reached the equilibrium stage. Additionally, the linear relationship between the calculated and experimental binding energy was fitted to further validate the result (Figure 3). The calculated data (Δ*G*_cal_, −43.58 to −24.43 kcal mol^−1^) is consistent with the experimental data (Δ*G*_exp_, −10.40 to −6.86 kcal mol^−1^) with high correlation coefficient *R*^2^ (0.92), suggesting that the trajectories from molecular dynamics were reliable.

**Figure 4:**
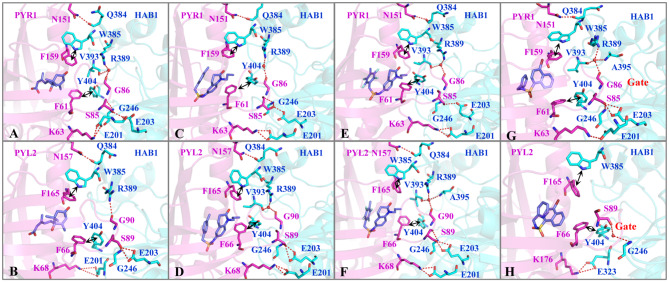
The interactions on the binding surface of PYLs and HAB1 after MD. The important residues of **(A,C,E,G)** PYR1 and **(B,D,F,H)** PYL2 are colored in magentas. The residues of HAB1 are colored in cyan. The ligands **(A,B)** ABA, **(C,D)** AM1, **(E,F)** AMF4, and **(G,H)** Pyrabactin are colored in blue.

The binding modes were further analyzed to study the interactions on the binding surface of HAB1 and PYR1/PYL2. As for agonists, the conservative ELE interactions were kept during MD, such as the hydrogen bond of Lys63/68-Glu201, Ser85/89-Gly246, Ser85/89-Glu203, and Asn151/157-Gln384 between PYR1/PYL2 and HAB1. Meanwhile, Gly86/90 of PYR1/PYL2 contacted with Arg389 of HAB1 through hydrogen bond or water bridge (Table S4). Furthermore, the *T*–π interaction between Phe61/66 of PYR1/PYL2 and Tyr404 of HAB1 as well as the π–π interaction between Phe159/165 of PYR1/PYL2 and Trp385 of HAB1 promoted the complexes formation (Figures 4A–G). Due to the conservative interactions on the binding surface, there is no absolute difference between ELE (−279.65 to −252.25 kcal mol^−1^) and VDW (−92.95 to −86.34 kcal mol^−1^) contribution in the systems binding with agonists (Table 2). However, the ELE contribution on the binding surface of PYL2 (Pyrabactin) and HAB1 dropped to −197.74 kcal mol^−1^, which induced their low binding affinity directly (Table 2). Based on the binding mode and the hydrogen bond monitoring result, this research found that the conserved hydrogen bonds were broken, such as the hydrogen bond of Lys68-Glu201, Gly86-Arg389, Ser89-Glu203, and Asn151-Gln384 between PYL2 and HAB1. Nonetheless, the Ser89 of PYL2 contacted with Gly246 of HAB1 through “water bridge,” in which a new hydrogen bond was formed between Lys176 of PYL2 and Glu323 of HAB1. On the other hand, it seems that the hydrophobic interaction between PYL2 (Pyrabactin) and HAB1 was no better than that of PYR1 (Pyrabactin)-HAB1 (Figure 4H). The π–π interaction between Phe165 of PYL2 and Trp385 of HAB1 was not stable as other systems, and the hydrophobic interactions between the gate of PYL2 and HAB1 were reduced.

**Table 2:** The binding free energy (kcal/mol) of HAB1 and PYLs (PYR1 and PYL2) complexed with ligands from calculation and experiment.

	**Δ*E*_**ele**_**	**Δ*E*_**vdw**_**	**Δ*E*_**MM**_**	**Δ*G*_**solv**_**	**ΔG_**cal**_**	**IC_**50**_ (nM)**	**Δ*G*_**exp**_**
PYR1-ABA	−276.76	−92.95	−369.71	330.44	−39.27	307.0	−8.9
PYL2-ABA	−266.80	−91.13	−357.94	316.92	−41.02	151.0	−9.4
PYR1-AM1	−279.65	−91.12	−370.78	328.87	−41.90	103.0	−9.6
PYL2-AM1	−252.25	−89.87	−342.12	301.48	−40.64	267.0	−9.0
PYR1-AMF4	−252.33	−90.69	−343.02	300.04	−42.98	119.1	−10.2
PYL2-AMF4	−267.41	−86.34	−353.75	310.17	−43.58	85.7	−10.4
PYR1-Pyrabactin	−257.12	−89.83	−346.95	310.16	−36.79	656.0	−8.5
PYL2-Pyrabactin	−197.74	−79.48	−277.22	252.80	−24.43	>10,000	−6.9

**Figure 5:**
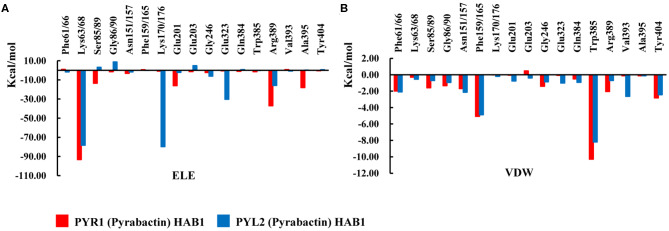
The comparison of the interactions of HAB1 and PYR1/PYL2 complexed with Pyrabactin. The **(A)** electronic (ELE) and **(B)** van der Waals (VDW) contributions of the important residues on the binding surface of PYR1 and PYL2 are shown in red and blue.

To reveal protein–protein interactions influenced by Pyrabactin from the energy aspect, the energy decompositions of amino acid residues on the binding surface of HAB1 and PYR1/PYL2 complexed with Pyrabactin were performed. In comparison to PYR1, the ELE contributions of Lys68, Ser89, Gly90 in PYL2 and Glu201, Glu203, and Arg389 in HAB1, induced by the loss of hydrogen bonds or water bridges, were reduced by 15.04, 16.61, 10.18, 13.81, 6.18, and 21.17 kcal mol^−1^, respectively (Figure 5A). On the other hand, the ELE contributions of Lys176 and Glu323 of PYL2 and HAB1 were improved from −0.82 and −0.56 kcal mol^−1^ to −79.77 and −30.31 kcal mol^−1^ because of the new hydrogen bond formed. The VDW contributions on the binding surface were also influenced: the contribution of Trp385 in HAB1, which formed π–π interaction with Phe165 of PYL2, was reduced by −2.09 kcal mol^−1^; the contributions of Ser89 and Gly90 on the gate were also lower than those with PYR1. The decomposition analysis of the binding energy was in accordance with the protein–protein interaction analysis results. From above, the distributions of Ser89 and Gly90 of PYL2 binding with Pyrabactin to ELE and VDW were both reduced significantly. These differences derived from the conformation of the gate determined by the Pyrabactin.

In short, the closed gate formed a better binding surface of HAB1 and formed more interactions with the downstream proteins (Figures 4G,H). This result is consistent with the gate–latch–lock mechanism underlying ABA signaling. When ABA binds to PYR1, the Pro88 of PYR1 on the gate moves toward the pocket to close the gate, whereas the Ser85 on the gate is flipped outward the cavity to contact with PP2Cs (Zhang et al., 2015). Meanwhile, Ser89 of PYL2 was reported as an important residue to form the tertiary complex (Yin et al., 2009). Furthermore, the mutations in PYL2 residues involving the formation of the gate compromise its ability to activate the reporter in response to ABA (Melcher et al., 2009). Therefore, gate-closed-induced ligands will be candidates for the ABA mimicking.

## Conclusion

In this study, we studied the selective activation mechanism of PYLs through the sequence alignment, molecular dynamics simulation, and binding free energy calculation methods. Even though the residues on the binding surface of PYR1 and PYL2 are conserved, the gate conformations of PYR1 and PYL2 induced by Pyrabactin are different. The Val114 in the pocket of PYL2 leads to a rotated binding model of Pyrabactin, which leads to an opened gate. This reduces the binding free energy of PYL2 and HAB1. The energy contribution changes of Lys63/68, Ser85/89, and Gly86/90 on the binding surface elucidate the selectivity of PYR1 and PYL2 complexed with Pyrabactin to HAB1. Therefore, the gate conformation influences the functions of PYLs directly. The results elucidate molecular determinants of the selectivity of PYLs and HAB1 interactions, which may provide new ideas for further agrochemical design and drought tolerance research.

## Data Availability Statement

The datasets generated for this study are available on request to the corresponding author.

## Author Contributions

G-FY and G-FH conceived of the research plan. J-FY, C-YY, and DW performed the project. G-FY, G-FH, J-FY, and C-YJ wrote the manuscript.

## Conflict of Interest

The authors declare that the research was conducted in the absence of any commercial or financial relationships that could be construed as a potential conflict of interest.

## Abbreviations

ABA: abscisic acid
PYR/PYL/RCARs: the Pyrabactin resistance/Pyrabactin resistance-like/regulatory component of ABA receptors
PP2Cs: protein phosphatase 2Cs
SnRK2s: SNF1-related protein kinase 2s
ELE: electrostatic
MD: molecular dynamics
MM/GBSA: the molecular mechanics/generalized born surface area
RMSD: the atomic root mean square deviations
STD: the standard deviation
VDW: van der Waals.
