# Supplementary material for: Molecular Determinants Elucidate the Selectivity in Abscisic Acid Receptor and HAB1 Protein Interactions
Source: Front Chem. 2020 Jun 4;8:425. doi: 10.3389/fchem.2020.00425 (PMC7287503; doi:10.3389/fchem.2020.00425)
Supplement: Supplementary file 1 [file Data_Sheet_1.PDF]

Supplementary Material

Molecular Determinants Elucidate the Selectivity in Abscisic Acid Receptor and HAB1 Protein Interactions

Jing-Fang Yang [c], Chun-Yan Yin [b], Di Wang [c], Chen-Yang Jia [c], Ge-Fei Hao [a,*], and Guang-Fu Yang [c,d,e,*]

[a]State Key Laboratory Breeding Base of Green Pesticide and Agricultural Bioengineering, Key Laboratory of Green Pesticide and Agricultural Bioengineering, Ministry of Education, Research and Development Center for Fine Chemicals, Guizhou University, Guiyang 550025, P. R. China; [b]School of Life Science, Wuchang University of Technology, 18 Jiangxia Road, Wuhan,430223, Hubei Province, P. R. China [c]Key Laboratory of Pesticide & Chemical Biology, Ministry of Education, College of Chemistry, Central China Normal University, Wuhan 430079, P.R.China; [d]International Joint Research Center for Intelligent Biosensor Technology and Health, Central China Normal University，Wuhan, 430079, China; [e]Collaborative Innovation Center of Chemical Science and Engineering, Tianjin 300072, P.R.China

Correspondence:

Ge-Fei Hao, Ph.D.

Professor

State Key Laboratory Breeding Base of Green Pesticide and Agricultural Bioengineering

Guizhou University

Huaxi Road

Guiyang 550025, P. R. China

E-mail: gefei_hao@foxmail.com

---

*To whom correspondence should be addressed. *E-mail:* gfyang@mail.ccnu.edu.cn; gefei_hao@foxmail.com

Table S1 The systems and timescales for molecular dynamics simulations.

Receptor	PDB ID	Ligand	Conformation[§]	Time (ns)
PYR1	3QN1	Pyrabactin[*]	Conf1	8
PYR1	3QN1	Pyrabactin[#]	Conf2	8
PYL2	3NS2	Pyrabactin[#]	Conf1	8
PYL2	3NS2	Pyrabactin	Conf2	8
PYR1-HAB1	3QN1	ABA	-	8
PYL2-HAB1	3KB3	ABA	-	8
PYR1-HAB1	3QN1	AM1[*]	-	8
PYL2-HAB1	4LA7	AM1	-	8
PYR1-HAB1	3QN1	AMF4[*]	-	8
PYL2-HAB1	5VSR	AMF4	-	8
PYR1-HAB1	3QN1	Pyrabactin[*]	Conf1	8
PYL2-HAB1	3NS2, 3QN1	Pyrabactin	Conf2	8

[*]The conformations of ligands generated from docking; [#]The conformations of ligands originated from superimposition; [§]The conformations of Pyrabactin: Conf1 was the conformation of it in PYR1 based on docking, Conf2 was the conformation of it in PYL2 from the crystal structure.

Table S2 The binding free energy (kcal/mol) of Pyrabactin in Conf1 and Conf2 and PYLs (PYR1 and PYL2) for per nanosecond in the last 6 nanoseconds.

PYR1 (Conf1)	ΔE_{ele}	ΔE_{vdw}	ΔE_{MM}	ΔG_{solv}	ΔG_{cal}
1	-15.15	-39.17	-54.32	32.07	-22.25
2	-14.94	-39.57	-54.51	33.81	-20.70
3	-16.37	-40.15	-56.52	36.46	-20.06
4	-17.91	-39.68	-57.59	40.15	-17.44
5	-16.62	-40.05	-56.67	36.00	-20.67
6	-16.10	-39.88	-55.98	36.65	-19.33
average	-16.18	-39.75	-55.93	35.86	-20.08
PYR1 (Conf2)	ΔE_{ele}	ΔE_{vdw}	ΔE_{MM}	ΔG_{solv}	ΔG_{cal}
1	-17.94	-40.37	-58.31	39.60	-18.71
2	-18.76	-39.53	-58.29	42.22	-16.07
3	-22.51	-39.57	-62.08	42.95	-19.13
4	-21.81	-39.04	-60.85	40.81	-20.04
5	-18.29	-38.82	-57.11	40.01	-17.10
6	-21.41	-39.71	-61.12	43.45	-17.67
average	-20.12	-39.51	-59.63	41.51	-18.12
PYL2 (Conf1)	ΔE_{ele}	ΔE_{vdw}	ΔE_{MM}	ΔG_{solv}	ΔG_{cal}
1	-15.83	-34.64	-50.47	45.31	-5.16
2	-16.03	-36.78	-52.81	45.49	-7.32
3	-16.96	-36.65	-53.61	47.26	-6.35
4	-14.86	-36.55	-51.41	43.73	-7.68
5	-13.01	-36.05	-49.06	42.52	-6.54
6	-13.33	-36.02	-49.35	41.49	-7.86
average	-15.00	-36.12	-51.12	44.30	-6.82
PYL2 (Conf2)	ΔE_{ele}	ΔE_{vdw}	ΔE_{MM}	ΔG_{solv}	ΔG_{cal}
1	-22.69	-38.92	-61.61	46.31	-15.30
2	-22.47	-37.77	-60.24	45.82	-14.42
3	-23.54	-36.54	-60.08	46.73	-13.35
4	-22.57	-35.04	-57.61	45.19	-12.42
5	-25.35	-36.91	-62.26	49.81	-12.45
6	-24.79	-37.38	-62.17	50.15	-12.02
average	-23.57	-37.09	-60.66	47.34	-13.33

Table S3 The calculated binding free energy (kcal/mol) of HAB1 and PYLs (PYR1 and PYL2) complexed with ligands per nanosecond in the last 6 nanoseconds.

PYR1-ABA	ΔE_{ele}	ΔE_{vdw}	ΔE_{MM}	ΔG_{solv}	ΔG_{cal}
1	-276.64	-95.15	-371.79	331.86	-39.93
2	-268.89	-93.32	-362.21	321.52	-40.69
3	-280.33	-92.59	-372.92	332.17	-40.75
4	-275.47	-94.53	-370.00	329.40	-40.60
5	-273.26	-91.91	-365.17	330.15	-35.02
6	-285.97	-90.19	-376.16	337.54	-38.62
average	-276.76	-92.95	-369.71	330.44	-39.27
PYL2-ABA	ΔE_{ele}	ΔE_{vdw}	ΔE_{MM}	ΔG_{solv}	ΔG_{cal}
1	-254.45	-94.91	-349.36	308.27	-41.09
2	-257.05	-92.48	-349.52	308.63	-40.89
3	-252.84	-90.50	-343.34	302.87	-40.47
4	-267.25	-88.49	-355.75	314.34	-41.41
5	-293.58	-89.67	-383.25	343.78	-39.47
6	-275.64	-90.75	-366.39	323.62	-42.77
average	-266.80	-91.13	-357.94	316.92	-41.02
PYR1-AM1	ΔE_{ele}	ΔE_{vdw}	ΔE_{MM}	ΔG_{solv}	ΔG_{cal}
1	-258.54	-89.30	-347.84	302.86	-44.98
2	-273.29	-89.27	-362.56	321.24	-41.32
3	-284.47	-91.34	-375.82	332.60	-43.22
4	-288.48	-93.07	-381.55	340.68	-40.87
5	-296.61	-94.34	-390.95	349.86	-41.09
6	-276.51	-89.42	-365.93	326.00	-39.93
average	-279.65	-91.12	-370.78	328.87	-41.90
PYL2-AM1	ΔE_{ele}	ΔE_{vdw}	ΔE_{MM}	ΔG_{solv}	ΔG_{cal}
1	-239.33	-90.77	-330.10	291.85	-38.26
2	-244.76	-90.46	-335.22	295.38	-39.84
3	-266.47	-88.81	-355.28	312.15	-43.13
4	-253.67	-90.27	-343.95	302.28	-41.67
5	-242.72	-89.89	-332.60	292.05	-40.56
6	-266.56	-89.03	-355.59	315.19	-40.40
average	-252.25	-89.87	-342.12	301.48	-40.64
PYR1-AMF4	ΔE_{ele}	ΔE_{vdw}	ΔE_{MM}	ΔG_{solv}	ΔG_{cal}
1	-232.04	-90.93	-322.97	279.93	-42.03
2	-241.99	-91.86	-333.85	289.34	-44.51
3	-269.01	-91.11	-360.12	317.17	-42.95
4	-258.88	-90.63	-349.51	306.18	-43.33
5	-244.87	-90.25	-335.12	294.03	-41.08

6	-267.20	-89.37	-356.56	312.62	-43.95
average	-252.33	-90.69	-343.02	300.04	-42.98
PYL2-AMF4	ΔE_{ele}	ΔE_{vdw}	ΔE_{MM}	ΔG_{solv}	ΔG$_{cal}$
1	-269.00	-84.62	-353.62	310.41	-43.21
2	-263.48	-83.98	-347.46	305.28	-42.18
3	-283.51	-85.06	-368.57	325.83	-42.74
4	-266.20	-87.54	-353.75	310.26	-43.49
5	-250.51	-88.60	-339.11	295.17	-43.94
6	-271.74	-88.23	-359.97	314.05	-45.91
average	-267.41	-86.34	-353.75	310.17	-43.58
PYR1--Pyrabactin	ΔE_{ele}	ΔE_{vdw}	ΔE_{MM}	ΔG_{solv}	ΔG$_{cal}$
1	-272.78	-88.15	-360.93	324.59	-36.34
2	-267.68	-89.14	-356.82	320.35	-36.46
3	-274.03	-90.70	-364.72	325.58	-39.15
4	-266.24	-89.96	-356.20	319.49	-36.71
5	-241.81	-91.17	-332.98	296.79	-36.20
6	-220.20	-89.87	-310.07	274.17	-35.89
average	-257.12	-89.83	-346.95	310.16	-36.79
PYL2--Pyrabactin	ΔE_{ele}	ΔE_{vdw}	ΔE_{MM}	ΔG_{solv}	ΔG$_{cal}$
1	-176.70	-78.86	-255.57	228.06	-27.50
2	-197.46	-81.86	-279.32	254.40	-24.92
3	-207.75	-80.01	-287.76	263.70	-24.06
4	-178.09	-76.84	-254.93	233.00	-21.93
5	-197.43	-79.29	-276.72	253.64	-23.09
6	-228.99	-80.05	-309.04	283.98	-25.06
average	-197.74	-79.48	-277.22	252.80	-24.43

Table S4 The hydrogen analysis of the binding surface of HAB1 and PYLs (PYR1 and PYL2) complexed with different ligands in the last 3 nanoseconds.

H-bond acceptor	H-bond donor	PYR1 (ABA)-HAB1		PYL2 (ABA)-HAB1	
		Occupied (%)	Distance (Å)	Occupied (%)	Distance (Å)
HAB1: Gln384@O	PYLs: Asn151/157@HD	80.2	2.91	99.6	2.87
PYLs: Gly86/90@O	H_2O@H	88.2	2.89	—	—
H_2O@O	HAB1: Arg389@HH	89.1	2.93	—	—
HAB1: Val393@O	H_2O@H	86.9	2.93	—	—
PYLs: Gly86/90@O	HAB1: Arg389@HH	—	—	85.3	3.14
PYLs: Ser85/89@OG	HAB1: Gly246@H	99.9	2.95	99.9	3.00
HAB1: Glu203@OE	PYLs: Ser85/89@HG	99.0	2.68	99.9	3.11
HAB1: Glu201@OE	PYLs: Lys63/68@HZ	99.9	2.86	99.9	2.86
H-bond acceptor	H-bond donor	PYR1 (AM1)-HAB1		PYL2 (AM1)-HAB1	
		Occupied (%)	Distance (Å)	Occupied (%)	Distance (Å)
HAB1: Gln384@O	PYLs: Asn151/157@HD	99.5	2.9	89.6	2.89
PYLs: Gly86/90@O	H_2O@H	99.9	2.81	98.3	2.86
H_2O@O	HAB1: Arg389@HH	93.6	2.95	99.9	2.84
HAB1: Val393@O	H_2O@H	—	—	89.6	2.85
PYLs: Ser85/89@OG	HAB1: Gly246@H	99.9	2.94	99.9	2.93
HAB1: Glu203@OE	PYLs: Ser85/89@HG	99.9	2.62	99.9	2.62
HAB1: Glu201@OE	PYLs: Lys63/68@HZ	99.9	2.84	95.8	3.17
H-bond acceptor	H-bond donor	PYR1 (AMF4)-HAB1		PYL2 (AMF4)-HAB1	
		Occupied (%)	Distance (Å)	Occupied (%)	Distance (Å)
HAB1: Gln384@O	PYLs: Asn151/157@HD	99.8	2.88	99.5	2.88

PYLs: Gly86/90@O	H_2O@H	90.3	2.84	99.9	2.79
H_2O@O	HAB1: Arg389@HH	97.1	2.91	96.2	2.89
HAB1: Val393@O	H_2O@H	95.1	3.00	99.1	2.81
H_2O@O	HAB1: Ala395@H	—	—	86.1	3.07
PYLs: Ser85/89@OG	HAB1: Gly246@H	99.9	3.03	98.4	2.98
HAB1: Glu203@OE	PYLs: Ser85/89@HG	97.2	2.68	96.6	2.70
HAB1: Glu201@OE	PYLs: Lys63/68@HZ	96.2	3.0	99.9	2.95

H-bond acceptor	H-bond donor	PYR1 (Pyrabactin)-HAB1		PYL2 (Pyrabactin)-HAB1	
		Occupied (%)	Distance (Å)	Occupied (%)	Distance (Å)
HAB1: Gln384@O	PYLs: Asn151/157@HD	98.6	2.85	—	—
PYLs: Gly86/90@O	H_2O@H	99.9	2.74	—	—
H_2O@O	HAB1: Arg389@HH	99.8	2.88	—	—
HAB1: Val393@O	H_2O@H	99.6	2.73	—	—
H_2O@O	HAB1: Ala395@H	95.2	3.16	—	—
PYLs: Ser85/89@OG	HAB1: Gly246@H	99.9	3.10	—	—
PYLs: Ser85/89@O	H_2O@H	—	—	88.5	3.07
H_2O@O	HAB1: Gly246@H	—	—	79.2	3.30
HAB1: Glu203@OE	PYLs: Ser85/89@HG	99.2	2.88	—	—
HAB1: Glu323@OE	PYLs: Lys170/176@HZ	—	—	99.9	2.97
HAB1: Glu201@OE	PYLs: Lys63/68@HZ	78.9	2.96	—	—

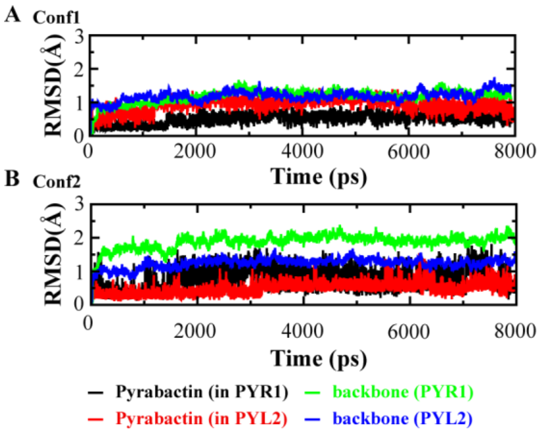

Figure S1 The RMSD of the CA atoms of receptors and heavy atoms of Pyrabactin with respect to the starting structure. The RMSDs of heavy atoms of Pyrabactin in the PYR1 and PYL2 respect to the starting conformation of it in the PYR1 (Conf1, A) and PYL2 (Conf2, B) complex structures are shown in black and red. The backbone RMSD of PYR1 (PDB ID: 3QN1) and PYL2 (PDB ID: 3NS2) are shown in green and blue.

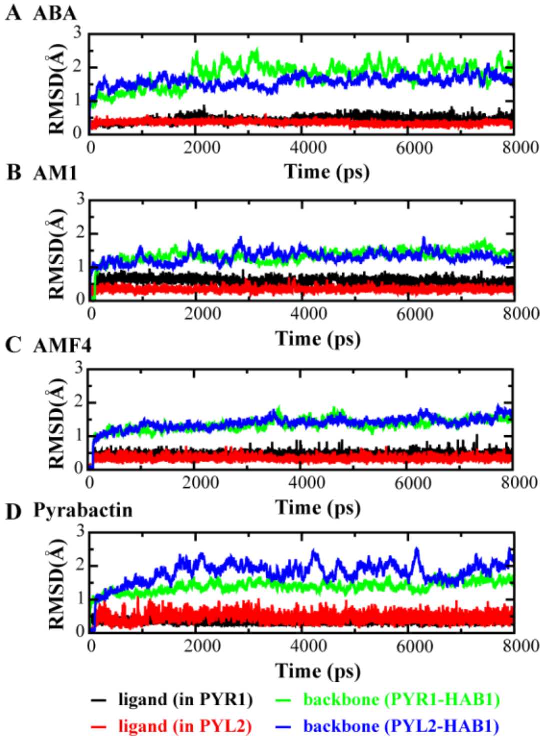

Figure S2 The RMSD of the CA atoms of receptors and heavy atoms of ligands with respect to the starting structure. The RMSD of heavy atoms of ligands in the PYR1 and PYL2 respect to the starting conformation of them in the complex structures are shown in

black and red. The backbone RMSD of PYR1 and PYL2 complexed with HAB1 are shown in green and blue. The systems and timescales for all molecular dynamics simulations were listed in the **Table S1**.
